# Prevention of Sunlight-Induced Cell Damage by Selective Blue-Violet-Light-Filtering Lenses in A2E-Loaded Retinal Pigment Epithelial Cells

**DOI:** 10.3390/antiox13101195

**Published:** 2024-10-01

**Authors:** Coralie Barrau, Mélanie Marie, Camille Ehrismann, Pauline Gondouin, José-Alain Sahel, Thierry Villette, Serge Picaud

**Affiliations:** 1 R&D Essilor International, 147 Rue de Paris, 94220 Charenton-Le-Pont, France; 2 Institut de la Vision, French National Institute of Health and Medical Research (INSERM), National Centre for Scientific Research (CNRS), Sorbonne Université, 75012 Paris, France; 3 Quinze-Vingts National Ophthalmology Hospital, French National Institute of Health and Medical Research (INSERM)-DGOS Clinical Investigation Center 1423, 28 Rue de Charenton, 75012 Paris, France; 4 Department of Ophthalmology, University of Pittsburgh School of Medicine and Medical Center, Pittsburgh, PA 15213, USA

**Keywords:** blue-violet light, oxidative stress, photoprotection, filter

## Abstract

Blue light accelerates retinal aging. Previous studies have indicated that wavelengths between 400 and 455 nm are most harmful to aging retinal pigment epithelia (RPE). This study explored whether filtering these wavelengths can protect cells exposed to broad sunlight. Primary porcine RPE cells loaded with 20 µM A2E were exposed to emulated sunlight filtered through eye media at 1.8 mW/cm^2^ for 18 h. Filters selectively filtering out light over 400–455 nm and a dark-yellow filter were interposed. Cell damage was measured by apoptosis, hydrogen peroxide (H_2_O_2_) production, and mitochondrial membrane potential (MMP). Sunlight exposure increased apoptosis by 2.7-fold and H_2_O_2_ by 4.8-fold, and halved MMP compared to darkness. Eye Protect System^TM^ (EPS) technology, filtering out 25% of wavelengths over 400–455 nm, reduced apoptosis by 44% and H_2_O_2_ by 29%. The Multilayer Optical Film (MOF), at 80% of light filtered, reduced apoptosis by 91% and H_2_O_2_ by 69%, and increased MMP by 73%, overpassing the dark-yellow filter. Photoprotection increased almost linearly with blue-violet light filtering (400–455 nm) but not with total blue filtering (400–500 nm). Selective filters filtering out 25% (EPS) to 80% (MOF) of blue-violet light offer substantial protection without affecting perception or non-visual functions, making them promising for preventing light-induced retinal damage with aesthetic acceptance for permanent wear.

## 1. Introduction

In addition to age, genetics, smoking, and diet, sunlight exposure is now widely established as a risk factor for retinal aging and Age-related Macular Degeneration (AMD) [1,2,3,4,5]. Specifically, the Chesapeake Bay Watermen study, involving 838 participants, demonstrated that patients with advanced AMD had significantly higher exposure to blue light over the preceding 20 years but equal exposure to UV light [4]. Similarly, the EUREYE study found a significant association between blue-light exposure and neovascular AMD in individuals with the lowest antioxidant levels (odds ratio; 1.09, 95% confidence interval 0.84 to 1.41) [5]. Studies on the risk of AMD progression following cataract surgery further support the hypothesis that blue-light exposure plays a role in AMD pathogenesis. Indeed, after cataract surgery with a clear UV-filtering intraocular lens (IOL), the increase in blue light reaching the retina is more than twice the proportion received by a 10-year-old retina and up to seven times more for a 70-year-old patient than before surgery (calculations derived from retinal transmittance data provided in CIE 203:2012) [6]. The AREDS studies also indicated that macular pigments, natural endogenous blue-light filters, can prevent or slow down AMD progression through dietary supplementation. Unfortunately, these pigments are less retained in aged patients.

As RPE cells accumulate lipofuscin [7], cellular sensitivity to light increases [8]. Lipofuscin contains high levels of A2E, a major retinoid fluorophore, which generates photodynamic damage under blue-light exposure, leading to RPE cell death [9,10,11,12,13,14,15,16,17,18]. This retinal aging was modeled by loading RPE cells with A2E, thereby demonstrating photodynamic damage characterized by the production of reactive oxygen species (ROS) such as hydrogen peroxide or by a loss of mitochondrial membrane potential [19,20,21,22,23,24,25,26]. Overexposure to blue light promotes ROS production, leading to retinal cell damage through oxidative stress and inflammatory responses [27]. Oxidative stress plays a crucial role in retinal aging and in AMD [28,29].

In our previous studies, we developed a custom-made LED-based device capable of delivering 10 nm wide illumination bands normalized to the corresponding daylight reaching the retina, accounting for natural eye media filtering. These studies enabled us to precisely define the most toxic spectral range for A2E-loaded RPE cells, between 400 and 455 nm, with a maximum between 415 and 455 nm [18,21]. While these studies confirmed the high blue-light toxicity in aging RPE cells, they did not demonstrate that removing part of this blue light in the solar spectrum could significantly prevent the degenerative process. We thus tested this hypothesis by developing a new custom-made device capable of mimicking the wideband visible sunlight spectrum reaching the retina, with the option to introduce filters in the optical path. Furthermore, we compared the protective effects of four optical lenses with progressive blue-violet filtering rates (400–455 nm) to those of a broadband blue-light filter, smoothly filtering wavelengths from 400 to 500 nm, simulating the transmittance of a classical yellow intraocular lens.

## 2. Materials and Methods

### 2.1. Cell Model

RPE cells were extracted from porcine eyes following local regulatory authorities and slaughterhouse veterinarians’ guidelines (agreement FR75105131), as previously described [18]. This procedure adheres to the European initiative for restricting animal experimentation, as no animals were killed specifically for our experimentation. Eyes were obtained from animals slaughtered daily for food production. Briefly, cells were seeded into a black 96-well plate and grown to confluence in the presence of serum. Confluent cells were then treated with A2E at 0 or 20 µM for 6 h in DMEM without serum. The absence of serum was required to remove any light-absorbing molecule in the culture medium and also to prevent fast cell division while not affecting cell viability. Subsequently, cells were washed twice with modified DMEM (medium without any photosensitizer such as phenol red, riboflavin, folic acid, or aromatic acids; Life Technologies, Carlsbad, CA, USA) and exposed to light for 18 h.

Starting at the A2E treatment step, all the experiments were conducted in darkness conditions under dim red light to restrict light exposure to the one from the controlled light system and avoid fluorescent or bioluminescent marker degradation.

### 2.2. Tailor-Made Adjustable Light Device

**Figure 1:**
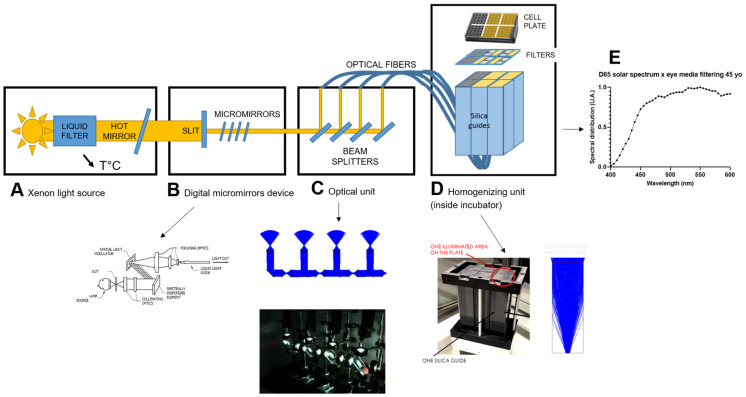
Tailored light set-up for simulating sunlight exposure at the retinal level. The light device consists of four units: (**A**) the lighting unit housing the light source; (**B**) the digital micromirror device which regulates both power and spectrum; (**C**) the optical unit separating the beam into four identical beams with the same optical power; and (**D**) the homogenizing unit. All units but the homogenizing unit are placed outside of the cell incubator to avoid heating and vibrations. The device enables exposure of a 96-well plate, divided into four sections of 16 wells each. Irradiance levels and homogeneity among the subdivision of the cell plate were monitored before and after each experiment, using a calibrated spectroradiometer. Additionally, light delivered by the custom-made device was continuously monitored using a calibrated photodiode. The current of the Xenon source was adjusted to stabilize the light level. The custom-made visible-light source can be spectrally adjusted to replicate the solar spectrum across 400–600 nm, weighted by eye media filtering (**E**).

We developed a purpose-made visible-light source that can be spectrally adjusted to mimic any type of light source over 400–600 nm, including artificial light sources and sunlight. The device comprises four units: (A) the lighting unit containing a 1000 W Xenon light source, (B) the digital micromirror device adjusting both the power and spectrum, (C) the optical unit separating the beam into four identical beams with the same optical power, and (D) the homogenizing unit inside the incubator (Figure 1). All units but the homogenizing unit are placed outside of the cell incubator to avoid heating and vibrations.

(A) The lighting unit contains a 1000 W Xenon source (Figure 1A). Xenon sources have a broad, continuous, and nearly flat spectrum over the visible range, which allow them to be easily spectrally adjusted. These sources are widely used as solar simulators. To avoid illuminating the cells with wavelengths outside of the visible range and to avoid overheating the optical system, two filters are used: a liquid filter which removes the infrared range between 1100 and 3000 nm, and a hot mirror which removes wavelengths between 780 and 1400 nm. Proper air circulation inside the optical system housing ensures a stable temperature for normal operation of the Xenon source.

(B) At the end of the optical path, the light is focused onto the 2 mm entrance slit of the digital micromirror device (Figure 1B). A spectrally dispersing element separates the light beam into its spectral components, which are reflected with a different angle depending on their wavelength. Each wavelength arrives on a specific range of micromirrors. The micromirrors can be switched on and off depending on the chosen spectrum. When they are switched on, the micromirror inclination reflects the light towards two focusing optics to be coupled into a liquid light guide. If the micromirrors are switched off, their inclination reflects the light towards an absorbing surface. The ranges of micromirrors switched on correspond to the exiting light spectrum. The number of micromirrors switched on for each range corresponds to the power of each wavelength.

(C) The fiber guides the beam to the optical unit which separates the beam into four different beams of the same optical power (Figure 1C). To do so, three beam splitters with specific transmission and reflection coefficients and a mirror are used. Successive collimations with achromatic lenses are carried out between each beam splitter to avoid optical power losses. The four beams are then coupled into four liquid light fibers that guide the light inside the incubator and the homogenizing unit.

(D) The homogenizing unit consists of parallelepiped rectangle silica guides which are 100 mm long and whose faces are the size of a 16-well subdivision of the cell plate, 35 × 35 mm^2^ (Figure 1D). The output beam of the optical fiber diverges significantly, with a numerical aperture of 0.59, and undergoes multiple reflections over the sides of the silica guides, ensuring a beam homogeneity over 35 × 35 mm^2^ at more than 90%.

The irradiance level and homogeneity between cell plate subdivisions were monitored before and after each experiment using a calibrated spectroradiometer, JAZ (Ocean Optics Inc, Dunedin, FL, USA) with a cosine corrector probe and calibrated in absolute irradiance. Also, light delivered by the Xenon source was continuously controlled by a calibrated photodiode. The current of the Xenon source was adjusted via a retroaction process to stabilize the light level.

### 2.3. Blue-Light-Filtering Optical Lenses

**Figure 2:**
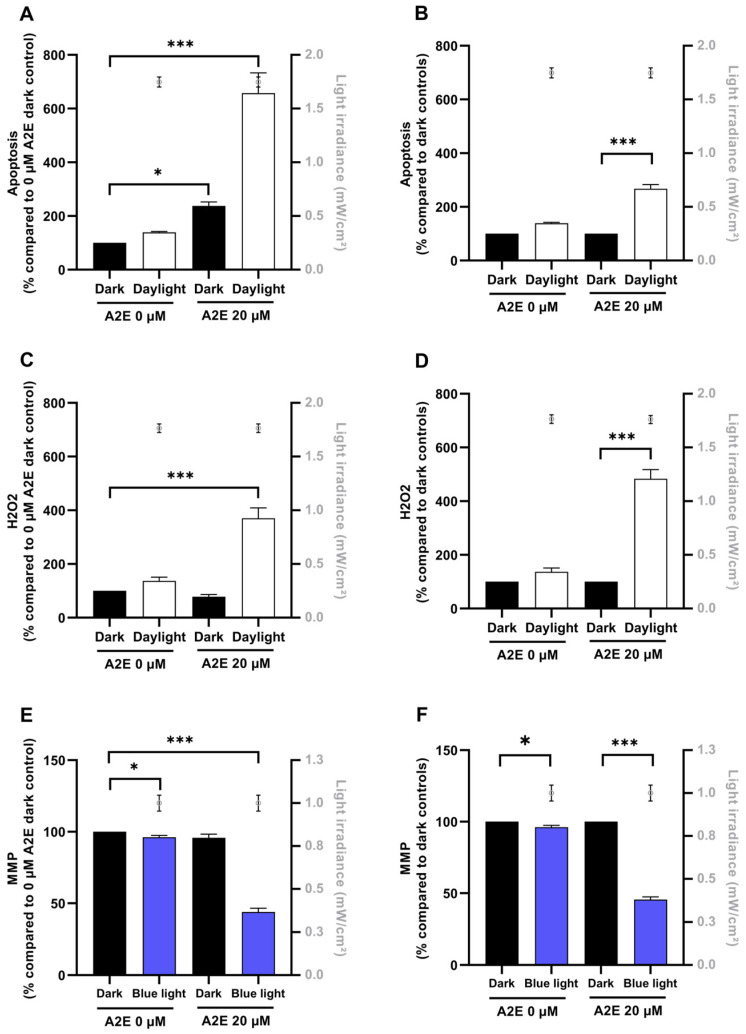
Evaluation of markers of cell damage in A2E-loaded cells exposed to simulated daylight. A2E-loaded RPE cells were exposed to simulated daylight at retinal level for 18 h before evaluation of apoptosis (*n* = 21 experiments; per experiment, 1 condition averaged on at least 12 wells). For each condition, applied light irradiances are expressed as mean +/− SEM in mW/cm^2^; with the grey symbol ο. (**A**,**B**), hydrogen peroxide (*n* = 8 experiments; per experiment, 1 condition averaged on at least 12 wells) (H_2_O_2_, **C**,**D**) and mitochondrial membrane potential (*n* = 4 experiments; 1 condition averaged on at least 12 wells) (MMP, **E**,**F**). Each marker was evaluated in darkness with and without A2E incubation and after light exposure in RPE cells incubated with 0 or 20 µM A2E. Data were either normalized to dark control with 0 µM A2E (**A**,**C**,**D**) or to dark control with 20 µM A2E (**B**,**D**,**F**). Data are expressed as mean +/− SEM. Two-way ANOVA with repeated measures and Tukey post hoc tests were used to compare variances between groups at each A2E concentration. Differences between sample and dark control were considered significant when *p* < 0.05 (*), or *p* < 0.001 (***).

Five types of optical filters were designed: the Eye Protect System^TM^ (EPS), a long-pass blue absorber (PUV), a narrow-band blue absorber (BA40), a Multilayer Optical Film (MOF), and a filter mimicking the transmittance of a yellow intraocular lens (Y-IOL) (Figure 2). These filters were characterized by the following parameters: blue-violet light reduction (BVC(B’) %), CUT_400–500 nm (%), reduction at 425 nm (%), reduction at 435 nm (%), Tv (%), T_460–500 nm (%), and a* and b* (Figure 2B).

Blue-violet light reduction (BVC(B’)) is the average reduction in light of the ophthalmic filter across 400–455 nm weighted by the blue hazard curve B’ derived from [18] (%). BVC(B’) is calculated as follows:

(1) $$BVC\left(B^{\prime }\right)=\frac{\int_{400}^{455\;nm}B^{\prime }\left(\lambda \right)T\left(\lambda \right)d\lambda }{\int_{400}^{455\;nm}B^{\prime }\left(\lambda \right)d\lambda }$$

CUT_400–500 nm is the average reduction across 400–500 nm (%).

CUT 425 nm is the reduction at 425 nm (%).

CUT 435 nm is the reduction at 435 nm (%).

Tv is the visual transmittance of the ophthalmic filter (%). It corresponds to the amount of visible light transmitted through the filter, weighted by the spectral sensitivity of the human eye under photopic conditions V(λ), as defined by ICI.

a* and b* are the cartesian coordinates of the CIELAB color space, calculated for a 10° observer or 1964 observer. a* is the quantity of red color (positive values) versus green color (negative values). b* is the quantity of yellow color (positive values) versus blue color (negative values).

a* and b* are calculated as follows:

(2) $$a^{*}=500\left(f\left(\frac{X}{X_{n}}\right)-f\left(\frac{Y}{Y_{n}}\right)\right)\;b^{*}=200\left(f\left(\frac{Y}{Y_{n}}\right)-f\left(\frac{Z}{Z_{n}}\right)\right)$$

where t = X/Xn, Y/Yn, or Z/Zn:

(3) $$f\left(t\right)=\left\{\begin{array}{ll} \sqrt[{3}]{t} & \mathrm{if}\;t>\delta^{3}\\ \frac{t}{3\delta^{2}}+\frac{4}{29} & \mathrm{otherwise} \end{array}\right.$$

X, Y, and Z describe the color stimulus and Xn, Yn, and Zn describe a specified white achromatic reference illuminant. The closer it gets to (a* = 0; b* = 0), the clearer the lens is. A standard lens without any blue-light filter exhibits a b* close to 1.

T_460–500 nm is the average transmittance of the ophthalmic filter between 460 and 500 nm (%), a range that corresponds to the highest sensitivity of melanopsin-based retinal ganglion cells (%), implied in the regulation of non-visual functions.

The EPS, PUV, BA40, and Y-IOL are absorptive filters. On the contrary, the MOF utilizes an interferential technology with self-assembled photonic crystals, creating a selective and high blue-violet reflection (Figure 2A). The EPS, PUV, BA40, and MOF selectively filter the blue-violet range (400–455 nm) with an increasing BVC(B’) of respectively 25%, 30%, 40% and 80% (Figure 2B). PUV is a long-pass absorber, filtering 100% of light below 408 nm, and then having a progressive increase in transmittance. The EPS, BA40, and the MOF are centered on 415–455 nm, which has been identified as the most harmful range in the 400–455 nm spectrum [17]. The MOF effectively filters 80% of wavelengths from 400 to 455 nm, with a total reduction from 420 to 435 nm, while reducing only 40% across the entire blue range, 400–500 nm. Y-IOL was conceived to superimpose the transmittance curve of a dark-yellow intraocular lens. Contrary to a MOF, Y-IOL is not a selective filter, as it filters out 55% of light across the whole blue band 400–455 nm, while removing 77% of blue-violet light. Consequently, this filter is very yellow orange in transmission (Figure 2A). The transmittance spectra of each filter were measured with the spectrophotometer Lambda900 (Perkin Elmer, Massachusetts, USA) from 400 to 600 nm at normal incidence (Figure 2C).

### 2.4. Light Exposure

To mimic physiological daylight, RPE cells were exposed for 18 h to a broadband visible-light spectrum between 400 and 600 nm, reproducing an average daylight spectrum with a correlated color temperature of 6500 K. The chosen normalized spectrum is the standard illuminant D65 (ISO/CIE 11664-2:2022|EN ISO/CIE 11664-2:2022) [30], onto which the eye media filtering of a 45-year-old person was applied (CIE 203:2012) [6] to consider the natural protection brought by the crystalline lens. Irradiance over 400–600 nm was defined as the best compromise to measure biological effects in exposure durations compatible with in vitro constraints, while keeping moderate and realistic light levels. Realistic light levels were obtained from irradiance measurements in Paris (GPS coordinates: 48.84983 and 2.372486) for an 8-month period, using the spectroradiometer JAZ (OceanOptics Inc., Dunedin, FL, USA) positioned inside the right eye of a 3D-printed mannequin head oriented downward at −15° to reproduce the head inclination of a walking person (supplementary from Marie et al., 2020 [31]). Spectral irradiances (mW/cm^2^) were measured with a 1 nm step and then integrated to compare to the emulated daylight. RPE cells were exposed to 1.8 ± 0.1 mW/cm^2^ over 400–600 nm, which corresponds to a 11-fold decrease for each 10 nm band compared to our previous study [18]. All irradiance values (mW/cm^2^) are reported as those measured at the cell level for in vitro studies and at the retinal level for real-life light measurements using the mannequin head.

Apoptosis and hydrogen peroxide were measured after exposure to 400–600 nm. Mitochondrial membrane potential, which is a highly specific and sensitive marker, was measured after exposure to the blue portion of the daylight-mimicking spectrum (400–500 nm). The irradiance applied for this blue spectral range (1.0 mW/cm ± 0.1 mW/cm^2^) corresponds proportionally to that of the same range within the broader 400–600 nm light range used for the other biomarkers.

Two 16-well areas of the 96-well plate were always maintained in darkness to control cell survival in the absence of irradiation (Figure 1D). The four remaining 16-well areas were exposed to light, without any filter or with the optical filters (unit size 17 × 35 mm^2^) randomly positioned. For each 16-well area, half were incubated with 0 µM A2E and the other half with 20 µM A2E. After light exposure, RPE cells were either kept in darkness for 6 h or directly characterized.

### 2.5. Apoptosis

After light exposure and a 6 h rest in darkness, apoptosis was assessed in A2E-loaded RPE cells using the ApoLive-Glo^TM^ assay kit (Promega, Madison, WI, USA) according to the manufacturer’s instructions and as previously described [18]. Measurements were performed on an Infinite M1000 microplate reader (Tecan, Männedorf, Switzerland). Apoptosis and viability were quantified according to the manufacturer protocol. The apoptosis level was defined as the ratio between caspase 3/7 and viability.

### 2.6. Hydrogen Peroxide

After light exposure, hydrogen peroxide (H_2_O_2_) was quantified in A2E-loaded RPE cells using the ROS-Glo™ H_2_O_2_ Assay kit (Promega, Madison, WI, USA) according to the manufacturer’s protocol. Briefly, cells were incubated with H_2_O_2_ substrate solution for 3 h before the end of the light exposure. Then, ROS-GLO Detection Solution was added at the end of the light exposure and incubated 20 min before luminescence reading on an Infinite M1000 microplate reader (Tecan, Männedorf, Switzerland).

### 2.7. Mitochondrial Membrane Potential

Mitochondrial membrane potential (MMP) was measured using the Mito-ID membrane potential cytotoxicity kit (Enzo Life Sciences, Farmingdale, NY, USA). Thirty minutes before the end of light exposure, carbonyl cyanide 3-chlorophenylhydrazone (CCCP, 4 µM) was added in a few wells to abolish the mitochondrial membrane potential as a positive control. At the end of light exposure, the Mito-ID dye was directly dispensed on cells and incubated for 30 min at room temperature. The dye fluorescence (ex 490 nm/em 590 nm) was quantified on a microplate reader (Infinite M1000, Tecan, Männedorf, Switzerland).

### 2.8. Photoprotection Potency of Filters

The in vitro photoprotection of each filter, reflecting its ability to mitigate the light-induced toxicity, was calculated as follows for each biomarker:

(4) $$Photoprotection=\frac{{Tox}_{NO\;FILTER}-{Tox}_{FILTER}}{{Tox}_{NO\;FILTER}}$$

Tox_FILTER_ is the light-induced toxicity measured with the filter compared to the 20 µM dark control.

Tox_NO FILTER_ is the light-induced toxicity measured without any filter compared to the 20 µM dark control.

### 2.9. Statistical Analysis

Data were expressed as mean ± SEM. Values were normalized to the respective dark control condition. For each experiment, each biomarker and each A2E concentration, the measures were averaged from 12 wells for darkness, from 12 wells for light without a filter, and from 4 wells for light with filters. All experiments were repeated at least four times for MMP and at least eight times for apoptosis and hydrogen peroxide quantifications with light and filters. Statistical analyses were performed using Statistica software version 12 (StatSoft, Tulsa, OK, USA). Without a filter: two-way ANOVA with repeated measures and Tukey post hoc tests were used to compare variances between groups at each A2E concentration. Differences between the sample and dark control were considered significant when *p* < 0.05 (*), *p* < 0.01 (**), or *p* < 0.001 (***). With a filter: all data were normalized to the dark control at 20 µM A2E. One-way ANOVA with repeated measures and post hoc Dunnett unilateral tests were used to compare the variance of all light-exposed groups with filters to the light condition without a filter at 20 µM of A2E. Differences between the sample and light without a filter were considered significant when *p* < 0.05 (*), *p* < 0.01 (**), or *p* < 0.001 (***). One-way ANOVA with repeated measures and Tukey post hoc tests were used to compare variances between light conditions with filters. Differences between filters were considered significant when *p* < 0.05 (#), *p* < 0.01 (##), or *p* < 0.001 (###). Photoprotection ratios obtained with the insertion of a filter were quantified as described above.

## 3. Results

### 3.1. Tailored Light Set-Up for Simulating Sunlight Exposure at the Retinal Level

We first developed a customized light source capable of replicating the solar spectrum reaching the retina after the natural filtering of the eye media across the range of 400–600 nm with a 5 nm resolution bandwidth. To meet these specifications, we opted for a high-power Xenon light source (Figure 1A), which was adjusted at the spectrum and light level in the visible range using a digital micromirror unit (Figure 1B). The emitted light was evenly distributed into four channels, with each channel illuminating 16 wells (35 × 35 mm^2^) of a 96-well plate following light equalization on the surface (Figure 1C). Holders were positioned within the optical pathway to allow the insertion of filters between the well plate and the light exiting the homogenizing unit, analogous to glasses positioned between the eyes and the light environment (Figure 1D).

The irradiance levels and homogeneity within each of the individual 16 well areas were monitored both before and after each light exposure using a calibrated spectroradiometer. Furthermore, the light output from the light source was continuously monitored by a calibrated photodiode.

The light was adjusted to expose A2E-loaded RPE cells to the sunlight spectrum received at the retinal level, maintaining an irradiance of 1.8 mW/cm^2^ over 400–600 nm for 18 h. This irradiance was selected based on real-life light measurements at the eye level using a 3D-printed mannequin head oriented downward at −15° to reproduce the head inclination of a walking person (supplementary from [31]). Outdoor measurements in Paris revealed irradiances ranging from 0.1 to 2.0 mW/cm^2^ in the 400–600 nm range, varying with the time of day, season, weather condition, etc. In summer, the average irradiance was 0.85 mW/cm^2^, regardless of the weather condition. Thus, an irradiance of 1.8 mW/cm^2^ over 400–600 nm for our experiments remains moderate, representing a cumulative effect rather than acute cell burns.

### 3.2. Sunlight-Induced Toxicity in A2E-Loaded RPE Cells

As in previous studies [18,21], retinal aging was modeled by loading RPE cells with the visual pigment derivative A2E at a concentration of 20 µM. When cells were treated with 20 µM A2E in darkness, apoptosis increased by 2.4-fold compared to the control condition with no A2E (*p* < 0.05) (Figure 2A). However, when investigating the molecular mechanisms underlying this cell death in darkness, we did not detect an increase in reactive oxygen species such as hydrogen peroxide (Figure 2C), nor a decrease in the mitochondrial membrane potential (Figure 2C). A significant but small decrease in mitochondrial membrane potential was observed after light exposure across the 400–500 nm range in non-loaded RPE cells compared to cells maintained in darkness (Figure 2E).

In contrast, light significantly affected all the measured parameters in A2E-loaded RPE cells (Figure 2). First, light exposure increased apoptosis in A2E-loaded cells by 6.6-fold compared to RPE cells without A2E (*p* < 0.001) and by 2.7-fold when compared to A2E-loaded RPE cells maintained in darkness for the same duration (*p* < 0.001) (Figure 2A,B). Light also increased the level of hydrogen peroxide in A2E-loaded cells by 4.8-fold compared to A2E-loaded RPE cells maintained in darkness (*p* < 0.001) (Figure 2D). Light also halved the mitochondrial membrane potential in A2E-loaded RPE cells as compared to A2E-loaded cells maintained in darkness (*p* < 0.001) (Figure 2E,F). These results confirm the effect of light toxicity on aging RPE cells modeled by loading RPE cell culture with A2E when applying a wide sunlight spectrum filtered by the eye media. This demonstrates the possibility of accurately characterizing the photoprotection potency of optical filters.

### 3.3. Blue-Light-Filtering Optical Lenses

**Figure 3:**
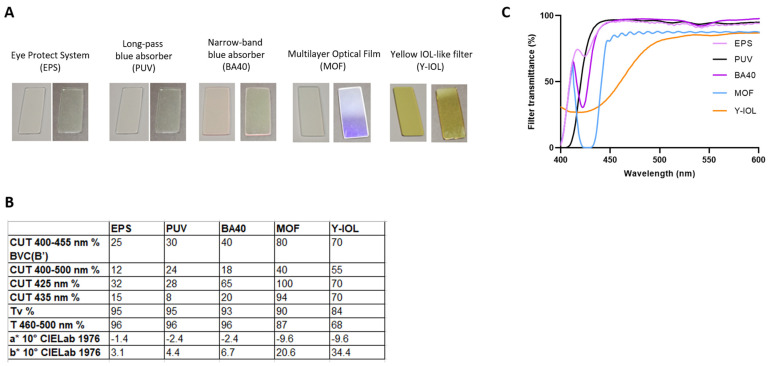
Characterization of five blue-light-filtering lenses. Filters were characterized by their appearance (**A**), their technology and optical properties (**B**), and their spectral transmittance across 400–600 nm (**C**). EPS and BA40 utilize narrow absorptive dyes, PUV functions as a longpass blue-light absorber, MOF is an interferential bandstop filter, and Y-IOL is a broadband absorptive filter. EPS, PUV, BA40, and MOF are selective blue-violet light filters (400–455 nm), whereas Y-IOL absorbs light smoothly across the entire blue-light range (400–500 nm).

In accordance with our previous studies demonstrating that light in the 400–455 nm range poses the highest toxicity to retinal cells, we conducted further investigations to determine whether filtering out this blue-violet light could mitigate solar light toxicity and how much filtering is required to achieve significant photoprotection. To achieve this, we developed four optical filters designed to selectively and variably filter the blue-violet light range while preserving essential circadian blue-turquoise light (notably 460–500 nm). These filters include the Eye Protect System^TM^ (EPS), a long-pass blue absorber (PUV), a narrow-band blue absorber (BA40), and the Multilayer Optical Film (MOF) (Figure 3). We also included a dark-yellow filter (Y-IOL), which unselectively attenuates 55% of the entire blue range, 400–500 nm, offering limited color perception and reducing essential circadian blue-turquoise light by 32% (Figure 3). The EPS, PUV, BA40, MOF, and Y-IOL exhibit a blue-violet light reduction across 400–455 nm at 25%, 30%, 40%, 80%, and 70%, respectively (Figure 3B and Figure 3C). Considering the entire blue-light range from 400 to 500 nm, the EPS, PUV, BA40, MOF, and Y-IOL exhibited average reductions at 12%, 24%, 18%, 40%, and 55%, respectively (Figure 3B and Figure 3C). Notably, as Y-IOL did not selectively filter blue-violet light, it showed a higher overall blue-light filtering across 400–500 nm compared to the MOF, while exhibiting a lower blue-violet light reduction across 400–455 nm. Interestingly, the EPS provided a modest average reduction in the entire blue-light spectrum across 400–500 nm (simple average of 12%), while presenting a significant selective blue-violet reduction over 400–455 nm, as indicated by BVC(B’) at 25%. Among the filters, the EPS and PUV offered the best balance between the attenuation of blue-violet light (25–30%), optimal clarity (Tv at 95%), and minimal residual tint (low a* and b*) (Figure 3B).

### 3.4. Photoprotection by Blue-Light-Filtering Lenses

**Figure 4:**
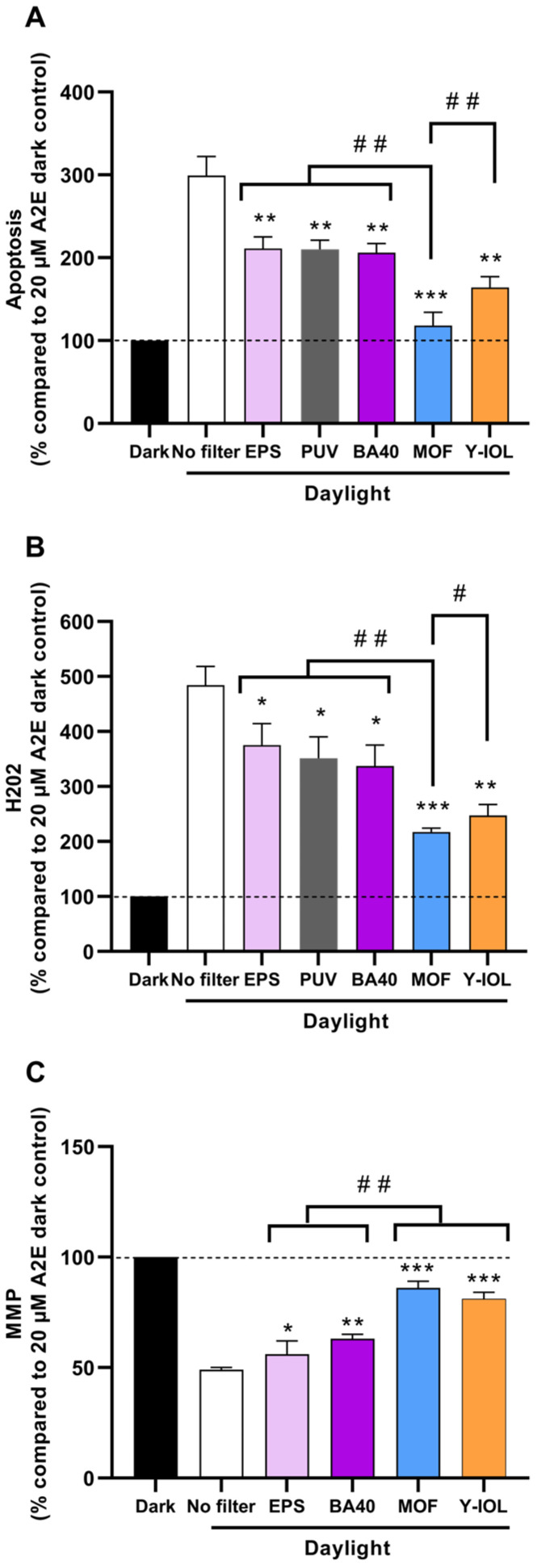
Photoprotection of filters in A2E-loaded cells exposed to simulated daylight. A2E-loaded RPE cells were exposed to simulated daylight at retinal level for 18 h without filter or with filter (EPS, PUV, BA40, MOF, or Y-IOL) before evaluation of apoptosis (**A**), hydrogen peroxide (H_2_O_2_, **B**), and mitochondrial membrane potential (MMP, **C**). Data are expressed as mean +/− SEM. Data are normalized to dark control with 20 µM A2E. One-way ANOVA with repeated measures and post hoc Dunnett unilateral test were used to compare variance of all light-exposed groups with filters to the light condition without filter at 20 µM of A2E. Differences between sample and light without filter were considered significant when *p* < 0.05 (*), *p* < 0.01 (**), or *p* < 0.001 (***). One-way ANOVA with repeated measures and Tukey post hoc tests were used to compare variances between light conditions with filters. Differences between filters were considered significant when *p* < 0.05 (#), or *p* < 0.01 (##).

To assess the protective efficacy of blue light filtering on A2E-loaded RPE cells, we exposed these cells to the solar light spectrum reaching the retina, as described previously, while incorporating light filters into the optical path. The optical filters evaluated in this study included the EPS, PUV, BA40, MOF, and Y-IOL. Compared to A2E-loaded RPE cells maintained in darkness, exposure to light without any filter resulted in a 3-fold increase in apoptosis (*p* < 0.001 for *n* = 8). This light-induced increase in apoptosis was significantly mitigated by 44% with both the Eye Protect System™ (EPS) (** *p* < 0.01) and PUV (** *p* < 0.01), by 47% with BA40 (** *p* < 0.01), by 69% with the Yellow IOL-like (Y-IOL) (** *p* < 0.01), and by 91% with the Multilayer Optical Film (MOF) filter (*** *p* < 0.001) (Figure 4A). Notably, when using the MOF filter, the level of apoptosis became no longer statistically different from that of cells kept in darkness (Figure 4A). While filtering less blue light across 400–500 nm than Y-IOL (40% vs. 55%), the MOF exhibited higher protection against light-induced apoptosis (## *p* < 0.01), which confirms the importance of light attenuation within the selective range from 400 to 455 nm (Figure 4A).

To further evaluate this photoprotection, we assessed the level of hydrogen peroxide (H_2_O_2_) in A2E-loaded RPE cells exposed to light in the presence of each optical filter. Compared to A2E-loaded RPE cells maintained in darkness, a 4.8-fold increase was observed in H_2_O_2_ levels after light exposure without any filter (*p* < 0.001). Similarly to apoptosis, a light-induced increase in H_2_O_2_ was mitigated by 30% with both the EPS (* *p* < 0.05) and PUV (* *p* < 0.05), by 38% with BA40 (* *p* < 0.05), by 62% with Y-IOL (** *p* < 0.01), and by 69% with the MOF (*** *p* < 0.001) (Figure 4B). The MOF exhibited higher protection against light-induced H_2_O_2_ compared to Y-IOL (# *p* < 0.05) (Figure 4B).

Finally, we also assessed the mitochondrial membrane potential (MMP), a recognized functional cellular marker affected during cell death. Simulated sunlight at the retinal level across 400–500 nm decreased the MMP by half in A2E-loaded RPE cells compared to A2E-loaded cells maintained in darkness (*p* < 0.001). This light-induced decrease in the MMP was partially mitigated by 14% with the insertion of the EPS (* *p* < 0.05), by 27% with the narrow-band blue absorber BA40 (** *p* < 0.01), by 63% with the non-selective dark-yellow filter (*** *p* < 0.001), and ultimately by 73% with the highly selective Multilayer Optical Film (MOF) (*** *p* < 0.001) (Figure 4C).

**Figure 5:**
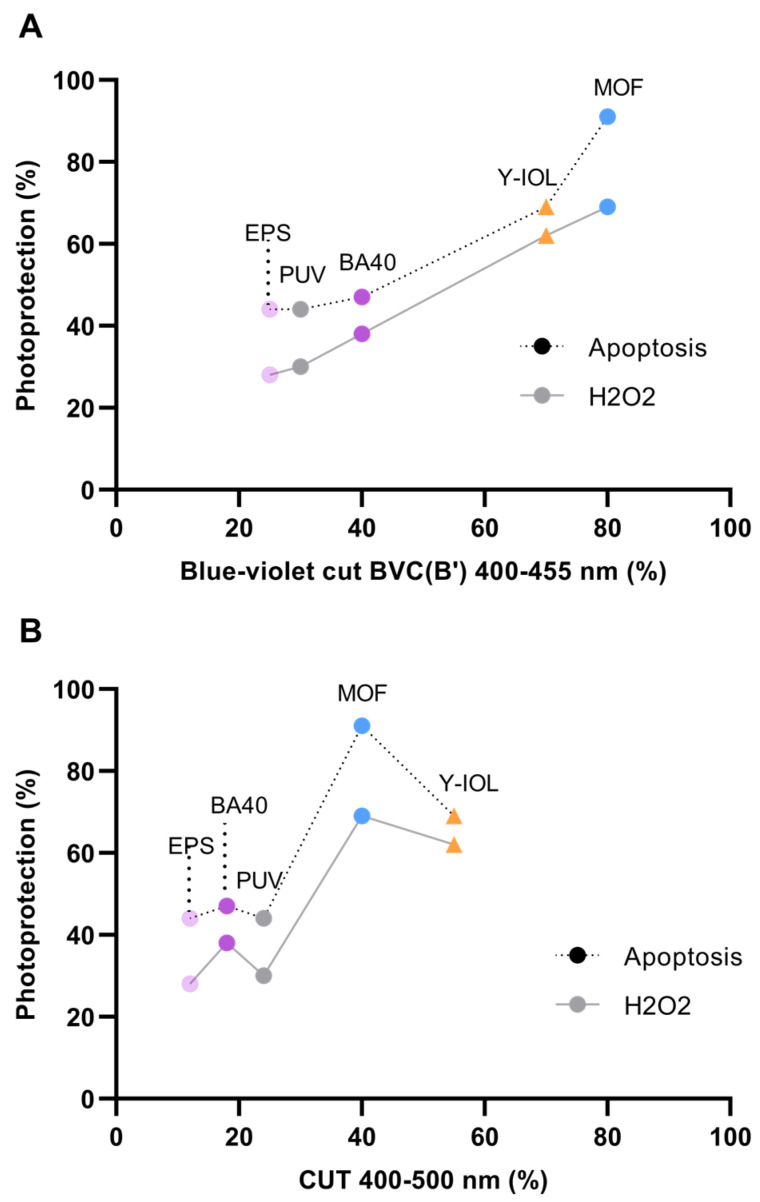
Photoprotection as a function of filtering properties. Photoprotection rates obtained with each filter against light-induced apoptosis (dotted lines in **A**,**B**) and H_2_O_2_ (gray lines in **A**,**B**) were plotted as a function of (**A**) their blue-violet reduction across 400–455 nm, expressed as BVC(B’), and (**B**) their average blue-light reduction across 400–500 nm. Each selective blue-violet filter is represented by a circle (EPS, PUV, BA40, and MOF), while Y-IOL is represented by a triangle to distinguish it as the only broadband filter. Each filter is depicted with a distinct color: EPS in light violet, PUV in gray, BA40 in vivid violet, MOF in blue, and Y-IOL in orange. (**A**) Increasing blue-violet reduction (400–455 nm) resulted in higher photoprotection, positioning MOF last after Y-IOL on the curves. (**B**) Conversely, an increasing average blue reduction (400–500 nm) did not consistently lead to increased photoprotection, as evidenced by MOF being positioned ahead of Y-IOL on the curve.

Y-IOL demonstrated stable photoprotection across the three tested biomarkers (Table 1), but the MOF consistently exhibited remarkable photoprotection efficacy, outperforming all other filters (Figure 5, Table 1). Specifically, the MOF’s highly selective and sharp filtering (80%) in the critical range of 400–455 nm effectively suppressed apoptosis induced by sunlight-mimicking exposure in A2E-loaded RPE cells. It is noteworthy that the MOF filters only 40% of light on average across the entire blue-light range of 400–500 nm, confirming that light-induced apoptosis and oxidative stress are primarily driven by the blue-violet range of 400–455 nm, particularly wavelengths between 420 and 435 nm (where the MOF filters out light completely) (Table 1, Figure 5).

Interestingly, doubling the reduction over 400–500 nm from 12% (EPS) to 24% (PUV) did not double the photoprotection efficacy, which was comparable for both filters (Table 1, Figure 5B). This result provides further confirmation that the blue-violet reduction, also comparable (25% for EPS and 30% for PUV), is a better metric for predicting the protection potency (Figure 5A). Additionally, the narrow-band blue absorber BA40, exhibiting a blue-violet light reduction at 40% while also having a limited average blue-light reduction across 400–500 nm, demonstrated a solid mitigation of light-induced retinal toxicity: photoprotection at 47% for apoptosis (slightly higher than the EPS and PUV), at 38% for H_2_O_2_, and at 27% for the MMP (Table 1, Figure 5).

The level of photoprotection obtained for H_2_O_2_ increased linearly with the blue-violet reduction BVC(B’) across 400–455 nm (R^2^ = 0.998) (Figure 5A), whereas it was not correlated with the average reduction across 400–500 nm (Figure 5B). The level of photoprotection obtained for apoptosis also increased with BVC(B’) (R^2^ = 0.957) (Figure 5A). Increasing blue-violet light reduction (400–455 nm) resulted in higher photoprotection for all biomarkers, positioning the MOF last after Y-IOL on the curves (Figure 5A). Conversely, an increasing average blue-light reduction (400–500 nm) did not consistently lead to increased photoprotection, as evidenced by BA40 being positioned ahead of PUV on the curves, and even more strikingly, the MOF being positioned ahead of Y-IOL despite its lower global filtering in the 400–500 nm range (Figure 5B). Indeed, note the change in position of the MOF and Y-IOL on the two graphs in Figure 5: the MOF provides the highest filtering in the 400–455 nm range, whereas Y-IOL exhibits the greatest filtering in the 400–500 nm range.

## 4. Discussion

Oxidative stress in retinal aging and AMD can be induced by multiple factors, including blue light from sunlight exposure. Strategies to reduce oxidative stress have been proposed as efficient treatments [32,33]. In this study, we developed an innovative and non-invasive method to prevent oxidative stress using optical filters.

Several studies have explored the cellular and molecular mechanisms of blue-light toxicity by modeling the aging retina with RPE cells loaded with A2E, a derivative of the visual pigment that accumulates in this epithelium with age [23,24,34,35,36]. While these studies demonstrated the toxicity of blue light, we refined the most harmful range as extending from 400 to 455 nm, with peak toxicity between 415 and 455 nm, applying 10 nm narrow-band light exposure [18,21]. Here, we inversely provide evidence that removing this portion of blue-violet light from the solar spectrum can mitigate light-induced toxicity.

Light exposure can be controlled accurately in in vitro studies, making them highly valuable for assessing the protective capabilities of optical filters. In 2011, for the first time, Zhou and Sparrow evaluated the protective effects of blue-light-absorbing filters containing perylene at various concentrations. They exposed ARPE cell lines to bright-blue light ranging from 410 to 450 nm, either for 20 min at 8 mW/cm^2^, i.e., 9.6 J/cm^2^, or for 30 min at 1 mW/cm^2^, i.e., 1.8 J/cm^2^ [37]. All filters provided protection, with the degree of protection dependent on dye concentration. This indicated that reducing the intensity of blue-violet light can mitigate cell damage. Similarly, Park and Jang confirmed that reducing blue-light exposure (430 ± 30 nm) at 9.4 mW/cm^2^ from an LED source for 10 min was more effective with brown- or gray-tinted filters compared to blue-tinted ones, as their percentage of filtered blue light was higher [38]. However, the narrow range of light exposure used in these studies did not accurately reflect wideband sunlight exposure. By developing a unique device that emulates the daylight spectrum at the retinal level, which is thoroughly monitored, we aimed to bridge this gap, providing a more representative assessment of filter efficacy. Our results suggest that selectively removing light in the 400–455 nm range with the MOF filter offers greater efficacy in terms of cell protection compared to broader filtering across 400–500 nm with Y-IOL. This aligns with our previous findings indicating that the 400–455 nm range is the most harmful part and thus should be targeted for removal in eyewear during daylight exposure.

Light-induced retinal damage involves various mechanisms depending on the light irradiance, duration, and spectrum [39]. Most in vitro studies on light-induced retinal damage apply very high light irradiances to shorten light exposure duration. For instance, Abdouh et al. delivered 100 mW/cm^2^ of blue light from 400 to 500 nm for 30 min [40], i.e., 180 J/cm^2^ across 400–500 nm, while Sparrow et al. delivered 246 mW/cm^2^ of white light (400–750 nm) for 20 min [41], i.e., 295 J/cm^2^ across 400–750 nm. For such acute light exposure, the observed mechanisms of cell damage and cell death may not represent the biological processes occurring in living conditions. Based on our outdoor light measurement campaign conducted in Paris for 8 months, we chose to illuminate cells with moderate light irradiance (1.8 mW/cm^2^). Indeed, this irradiance represents only twice the averaged summer irradiance received at the retinal level across the 400–600 nm range in Paris (0.85 mW/cm^2^) and enters in the natural irradiance range that we have measured, comprising between 0.1 and 2.0 mW/cm^2^. This moderate light irradiance (1.8 mW/cm^2^) over a wide visible range (400–600 nm) delivered for an extended exposure period (18 h) is expected to mirror chronic sunlight exposure patterns and the consecutive biological processes more accurately than high irradiance levels (~100 mW/cm^2^ or higher) for short durations (less than one hour). If we consider a flat light spectrum as a simplification hypothesis for light comparison purposes, the spectral light doses of previous studies, expressed in J/cm^2^/nm, were 1.8 J/cm^2^/nm and 0.8 J/cm^2^/nm, which correspond, respectively, to a 3-fold and a 1.4-fold increase in light irradiance with respect to our study (0.6 J/cm^2^/nm). The observed protection achieved by selectively filtering the 400–455 nm range may therefore represent cellular events occurring in aged patients exposed to sunlight over several consecutive days.

Filtering out blue light has already been reported to provide retinal protection both clinically, using blue-light-filtering IOL [35,40,41,42,43], and in vitro, using broadband blue-light-filtering lenses or IOL [34,36,44,45,46]. However, broadband filters have major limitations for permanent wear due to alterations in color vision and non-visual retinal functions (peak at 480 nm). In addition, such broadband ophthalmic filters generate a yellow tint that is widely rejected for aesthetic considerations by patients. The effect on non-visual functions is not desirable because it may affect all the circadian regulations, including day/night rhythms and mood control. In addition, it will also affect pupil constriction, increasing the amount of light entering the eye, thereby limiting or cancelling out the beneficial effect of the blue-light filtering. Our previous findings from in vitro studies favored more precise filtering in the blue-violet range, 400–455 nm [18,21], consistent with a recent ISO technical report [47]. The Eye Protect System™, PUV, BA40, and MOF ophthalmic filters were specifically designed to reduce the amount of blue-violet light entering the retina without affecting other visible wavelengths. This results in lenses that are minimally colored and selectively filter out harmful blue-violet light while leaving unaffected the blue-turquoise range that is essential for non-visual functions and circadian rhythms. The Eye Protect System™, PUV, and BA40 demonstrated measurable protection of RPE cells that have accumulated the aging lipofuscin fluorophore A2E. The MOF, filtering blue-violet at the highest level, significantly improved aesthetics and visual perception compared to conventional dark-yellow filters. The MOF consistently exhibited remarkable photoprotection efficacy, outperforming other filters, notably the conventional Y-IOL, making it a promising alternative for patients at risk for retinal diseases, AMD, or other cone dystrophies [31]. Our study underscores the importance of carefully designed in vitro models and light exposure devices for evaluating photoprotection. The findings highlight the importance of optimizing filter characteristics to effectively mitigate light-induced retinal damage, maximizing the blue-violet reduction, BVC(B’), and visual transmittance, Tv, while minimizing a* and b* and thus preserving vision, aesthetics, and circadian rhythms. The blue =0light filtering level across the whole blue range, 400 to 500 nm, is not well correlated with the in vitro photoprotection.

## 5. Conclusions

By developing a novel adjustable light device, we were able to evaluate the mitigation of light-induced damage provided by blue-light-filtering lenses on an in vitro model of retinal aging under physiological sunlight conditions. All blue-violet-selective filters (EPS, PUV, BA40, and MOF) demonstrated statistically significant potential for protecting RPE cells from light-induced damage based on their filtering properties in the 400–455 nm range, without compromising vision, aesthetics, and circadian rhythms. While the existing Eye Protect System™ and PUV filters offer protection, the sharp bandstop profile of the MOF filter demonstrates even greater prevention against apoptosis and oxidative stress in A2E-loaded RPE cells, outperforming a conventional dark-yellow filter (simulated by Y-IOL). Therefore, these findings underscore the importance of precise filtering in the blue-violet range to preserve circadian function and color vision while effectively mitigating light-induced damage, paving the way for advanced optical filters to promote enhanced eye health.

## 6. Patents

Patent by C.B., M.M., C.E., T.V., J.S., and S.P.

## Figures and Tables

**Table 1:** In vitro photoprotection provided by each optical filter for each biomarker.

	EPS	PUV	BA40	MOF	Y-IOL
Blue-violet cut BVC(B’) CUT 400–455 nm %	25	30	40	80	70
CUT 400–500 nm %	12	24	18	40	55
CUT 425 nm %	32	28	65	100	70
CUT 435 nm %	15	8	20	94	70
PHOTOPROTECTION % (calculated as described in Section 2)					
For apoptosis	44	44	47	91	69
For H_2_O_2_	28	30	38	69	62
For PMM	14	Not tested	27	73	63

## Data Availability

All data supporting the conclusions of the paper are available in the article and corresponding figures.
